# Gastric Volvulus, Pancreatic Volvulus, and Wandering Spleen: A Rare Emergency Triad Behind Acute Abdomen

**DOI:** 10.1002/ccr3.71007

**Published:** 2025-10-15

**Authors:** Parvaneh Layegh, Atiyeh Mahdavi Rafie, Armin Doostparast

**Affiliations:** ^1^ Department of Radiology, Faculty of Medicine Mashhad University of Medical Sciences Mashhad Iran; ^2^ Student Research Committee, Faculty of Medicine Mashhad University of Medical Sciences Mashhad Iran; ^3^ Eye Research Center Mashhad University of Medical Sciences Mashhad Iran

**Keywords:** acute abdominal pain, gastric volvulus, pancreatic volvulus, wandering spleen

## Abstract

Gastric volvulus is a rare but serious gastrointestinal disorder that can lead to obstructive complications, necessitating prompt diagnosis and management. Rarely, gastric volvulus coexists with scare conditions like pancreatic volvulus and wandering spleen as a triad, further complicating clinical management. Although the primary presentation is acute and severe abdominal pain, other findings may exist, including nausea, bilious vomiting, fever and chills, leukocytosis, and elevated liver or pancreatic enzymes. The diagnosis is usually confirmed by advanced radiologic techniques, mainly abdominal computed tomography scans. Early recognition and timely surgical correction are essential to prevent severe complications such as ischemia and necrosis. This paper presents two cases of gastric volvulus with pancreatic volvulus and wandering spleen, highlighting the importance of radiologic awareness and multidisciplinary management in such rare presentations.

## Graphical abstract

Coronal and 3D reconstruction illustrating the rare triad of gastric volvulus, pancreatic volvulus, and wandering spleen, highlighting abnormal organ positions and the importance of prompt diagnosis and surgical intervention in acute abdomen.

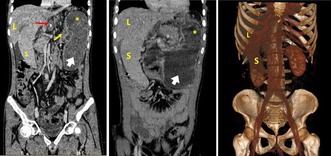

**Summary**

- Gastric volvulus, though rare, can present with life‐threatening complications, especially when coexisting with pancreatic volvulus and wandering spleen.
- Despite the rarity of this combination, it is an emergent abdominal condition and could lead to extremely serious complications if overlooked, highlighting the importance of early diagnosis and prompt subsequent surgical intervention.

## 1 Introduction

Gastric volvulus is an uncommon but potentially life‐threatening condition characterized by the abnormal twisting of the stomach along its axes, leading to obstruction and compromised blood flow (ischemia) [1, 2]. Initially described by Berti in 1866, this disorder poses significant diagnostic and therapeutic challenges due to its broad spectrum of clinical presentations [3, 4]. There is no gender predilection, and the highest incidence is observed in individuals in their fifth decade of life, with a secondary peak in infants under 1 year of age [3]. Predisposing factors include diaphragmatic defects such as hiatal or paraesophageal hernias, phrenic nerve dysfunction, congenital anomalies of the stomach or spleen, a history of bariatric surgery, and spinal deformities like kyphoscoliosis [2, 3, 5].

The clinical manifestations of gastric volvulus are highly variable, ranging from vague abdominal discomfort to acute abdominal emergencies, complicating timely diagnosis [2]. A classic presentation includes Borchardt's triad, which is present in 70% of the cases. It is defined as severe epigastric pain, unproductive retching, and the inability to pass a nasogastric tube [6, 7]. Etiologically, gastric volvulus can be classified as primary, resulting from congenital or acquired abnormalities in the gastric suspensory ligaments, or more commonly as secondary, associated with other anatomical defects such as diaphragmatic hernias [2, 5].

Intestinal obstruction is the primary complication, which can manifest acutely, recurrently, intermittently, or chronically [8]. Additionally, the risk of gastric strangulation can lead to severe outcomes, including gastric tissue necrosis, perforation, sepsis, hypovolemic shock, displacement of the other internal organs, and aspiration pneumonia [1, 5, 8, 9]. Diagnosis based on the history or physical examination is challenging, and the confirmation typically relies on radiological imaging, with plain radiographs, computed tomography (CT) scans, and upper gastrointestinal series playing crucial roles [5, 6]. Once diagnosed, surgical intervention is generally the preferred treatment approach [8, 10].

Given the significant morbidity and mortality rates linked to gastric volvulus, early recognition and prompt management are critical. This case series highlights two patients diagnosed with gastric volvulus, illustrating the diverse clinical presentations, diagnostic challenges, and treatment approaches associated with this rare condition.

## 2 Case 1

### 2.1 History/Examination

A 36‐year‐old man with no previous underlying disease presented to the emergency department with increasing peri‐umbilical abdominal pain for the past 2 days. The abdominal pain intensity was scored 9/10 while spreading all over the abdominal cavity over time, accompanied by frequent episodes of vomiting and the lack of defecation or gas passing, as stated by the patient. Upon admission, his general condition was ill with the following vital signs: blood pressure: 110/60, heart rate: 115, respiratory rate: 23, and oral temperature: 37.2°C. The abdominal examination revealed generalized distention, tenderness, and guarding, while the rectum contained feces in the rectal examination.

### 2.2 Methods (Differential Diagnosis, Investigations, and Treatment)

The upright and supine abdominal X‐rays were requested, demonstrating evidence of gastric distension despite no distention in the intestinal loops. Furthermore, a mixture of feces and gas was seen in the visible parts of the colon and rectum. Due to the patient's frequent vomiting, a nasogastric (NG) tube was inserted to resuscitate the patient. The laboratory test results were as follows: White Blood Cell (WBC) count: 13,200/μL, neutrophils: 75%, Hb: 14.2 g/dL, platelets: 254,000/μL, amylase: 1800 U/L, lipase: 600 U/L, ALT = 17 U/L, AST = 26 U/L, ALP = 125 U/L, Na = 134 mEq/L, K = 3.4 mEq/L. Given the patient's examinations and suspected obstruction, a contrast‐enhanced CT (CECT) scan was performed for further evaluation.

**FIGURE 1:**
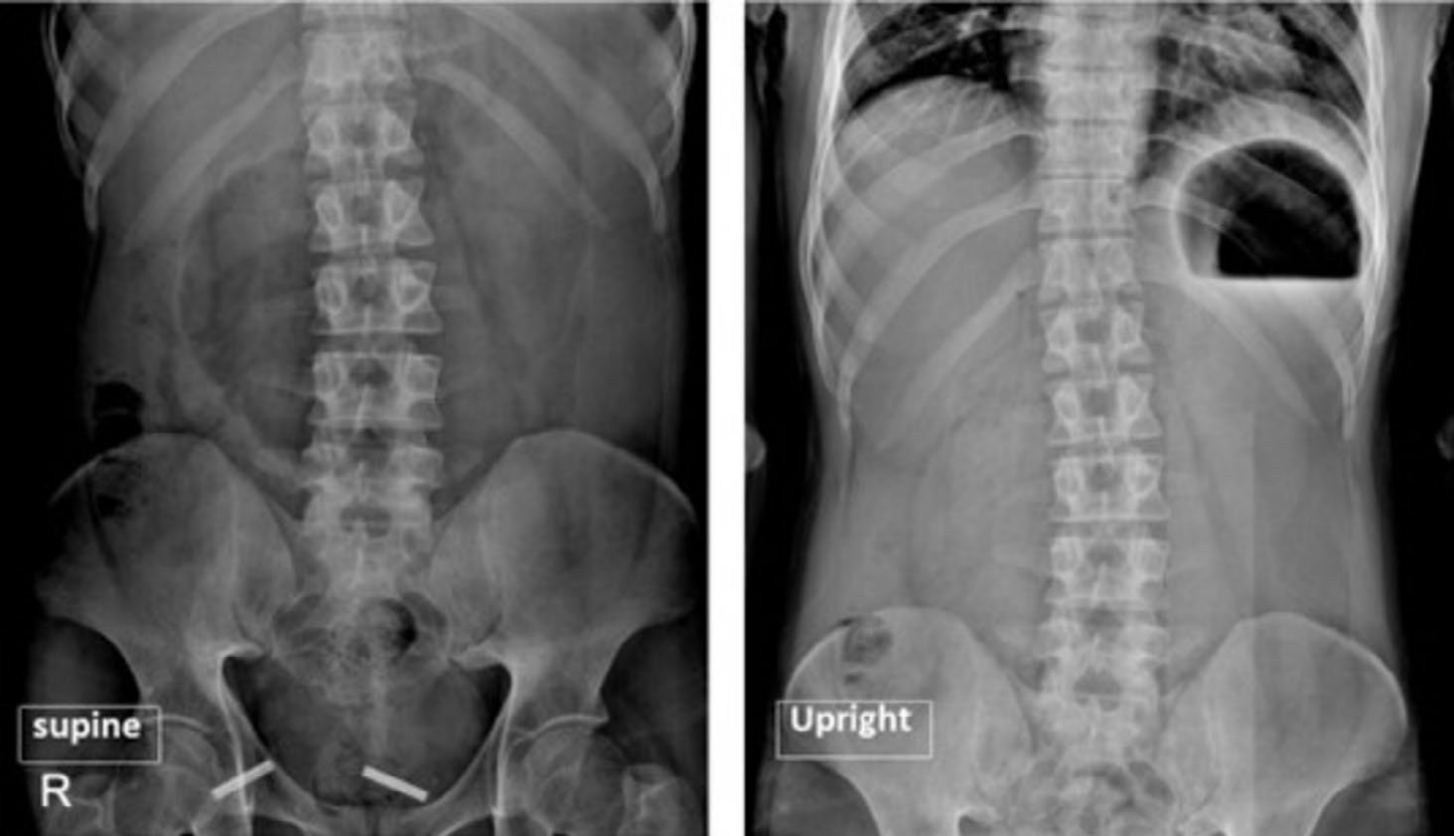
Supine (*left*) and upright (*right*) images show the distended stomach with an air‐fluid level.

**FIGURE 2:**
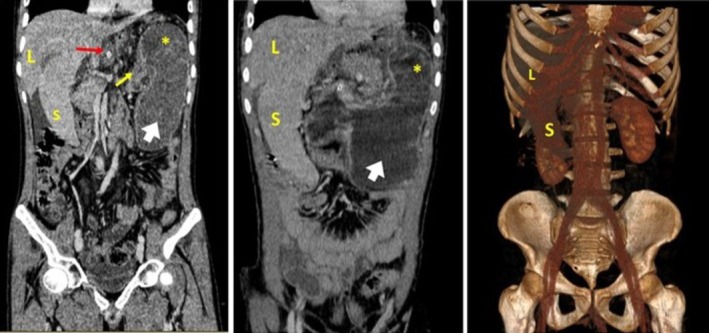
Coronal planes and 3D reconstruction image show malposition of the spleen (S) in the inferior and anterior position relative to the liver (L), consistent with the wandering spleen appearance. In addition, the distended stomach is evident as the fundus (*white arrow*) is positioned inferiorly. The antrum is (*yellow star*) on the superior left while the pylorus (*yellow arrow*) is positioned on the left, and the cardia (*red arrow, containing the NG tube*) on the right.

**FIGURE 3:**
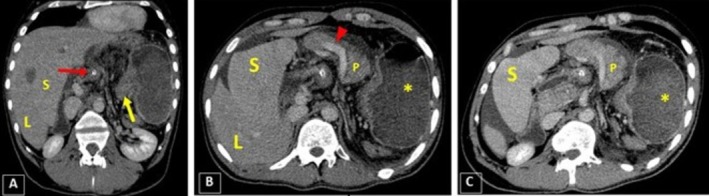
The coronal oblique image of CECT (A) shows pylorus (*yellow arrow*) on the left and cardia (*red arrow with the tip of NG tube*) on the right are at the same levels. In axial planes (B, C), there is also evidence of bulging in the pancreatic head and body (*P*), with surrounding inflammatory changes indicating pancreatitis. The spleen (*S*) is located on the right side, medial to the liver (*L*). Furthermore, the free fluid should be noted in the peri‐pancreatic, peri‐splenic, and peri‐hepatic spaces. Finally, the red arrow highlights the splenic vein.

The CT scan demonstrated evidence of severe gastric dilatation with an abnormal axis. The fundus was located in the inferior anterior position, while the antrum was in the superior posterior position on the left side. Moreover, the duodenum and the distal intestinal loops collapsed entirely, suggesting complete obstruction secondary to volvulus. Fortunately, no evidence of increased mucosal thickness or decreased enhancement indicative of ischemic changes was observed. In addition to the previous findings, the spleen was observed in an inferior anterior position on the right side relative to the liver, consistent with a wandering spleen. In addition, bulging of the pancreatic head with surrounding fat stranding was observed, along with the body and tail positioned medial to the spleen on the right side, suggesting a degree of pancreatic volvulus combined with pancreatitis. Notably, moderate free fluid was observed in both the abdominal and pelvic cavities. The right kidney hilum was also positioned laterally, suggesting a degree of renal malposition (Figures 1, 2, 3). The liver and other abdominopelvic organs appeared normal.

### 2.3 Conclusions and Results (Outcome and Follow‐Up)

Following the diagnosis of gastric volvulus and secondary complete obstruction, the patient was initially managed with aggressive intravenous (IV) fluid therapy, antibiotic injection (IV Metronidazole + ceftriaxone), Foley catheter insertion, and I/O monitoring. He was then immediately transferred to the operating room for surgery. After performing a midline laparotomy, approximately 300 cc of reactive fluid was suctioned from the abdomen. The spleen was found to be completely congested, unattached to its surrounding structures, and located near the stomach on the right side of the abdomen, contributing to the gastric volvulus and severe gastric distention. However, no signs of ischemia were observed in the stomach. Consequently, a splenectomy was performed, and the stomach was fixed at four points, followed by a gastrostomy. Regarding the pancreatic volvulus, no signs of ischemia or necrosis were observed intraoperatively. The pancreas appeared partially twisted, yet viable. Given the absence of obstructive or ischemic features, no direct surgical intervention on the pancreas was performed. The patient was then transferred to the postoperative ward for continued care. Postoperative management included close monitoring of pancreatic enzymes and supportive care, which resulted in clinical improvement. The recovery course proceeded without complications, and the patient was discharged in stable condition after 10 days.

## 3 Case 2

### 3.1 History/Examination

A 28‐year‐old man presented to the emergency department with a chief complaint of epigastric pain persisting for the past 5 days. The pain intensity has gradually increased while aggravating during movement since the last day, and increasing episodes of nausea and vomiting accompanied it. The vomiting was not projectile and initially contained semi‐digested food materials while turning to a dark yellow fluid without any solid materials in the latter episodes; however, these projections were not foul‐smelling. The patient has noticed decreased bowel habits recently, with no change in the bladder habits. A history of total colectomy due to Familial Adenomatous Polyposis (FAP), which was carried out 6 years ago, was also provided. Despite being mildly ill at the time of presentation, his vital signs were stable and normal (blood pressure: 115/70, heart rate: 97, respiratory rate: 19, and oral temperature: 36.8°C). However, the patient was evaluated to be mild to moderately dehydrated during the examinations and relatively nervous. During the abdominal examination, severe epigastric tenderness, mild to moderate distension, guarding, and rebound tenderness were recorded, all becoming more intense toward the epigastric region. The rectal examination was also found insignificant, as no abnormality was noted.

### 3.2 Methods (Differential Diagnosis, Investigations, and Treatment)

**FIGURE 4:**
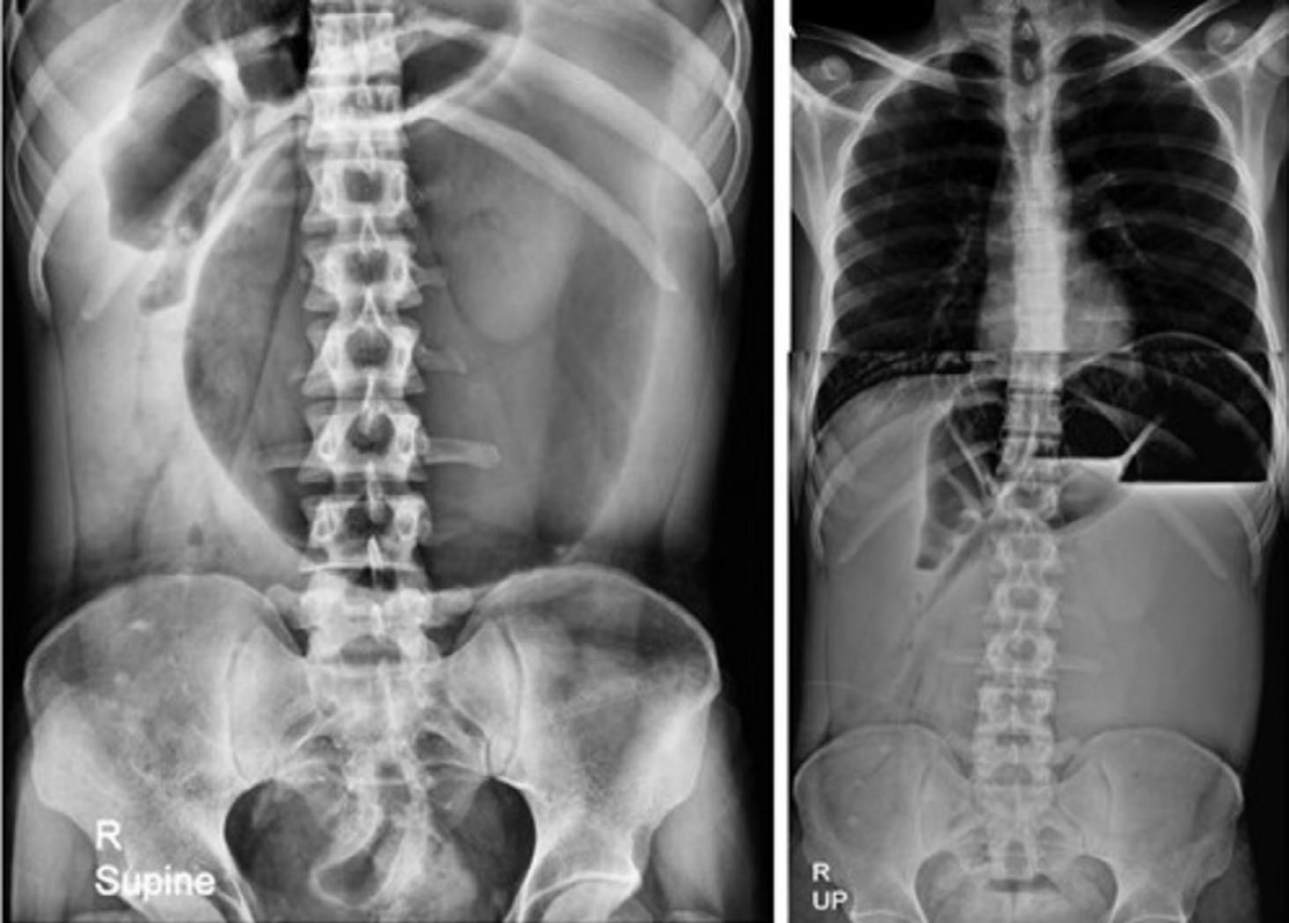
Supine (*left*) and upright (*right*) images showing a gastric distention with an air‐fluid level.

**FIGURE 5:**
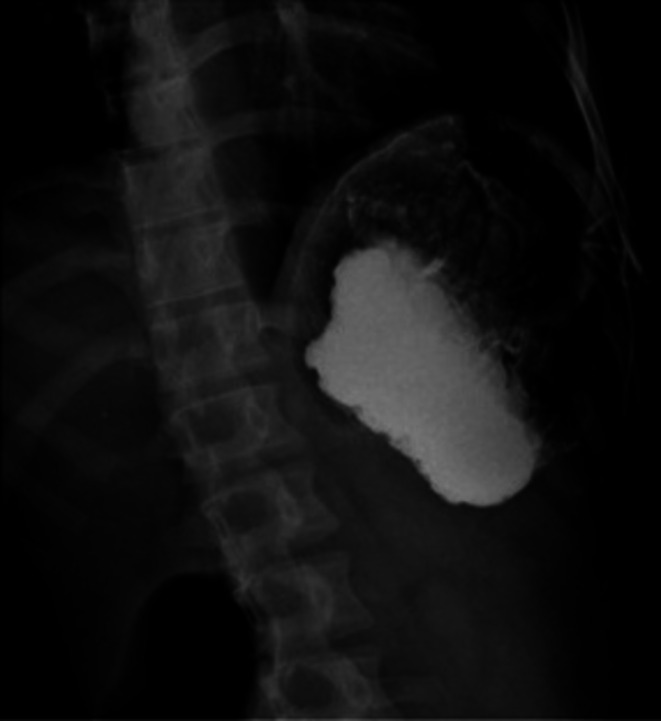
Left anterior oblique X‐ray after ingestion of water‐soluble positive contrast agents shows an abnormal gastric axis with an accumulation of contrast in the fundus.

**FIGURE 6:**
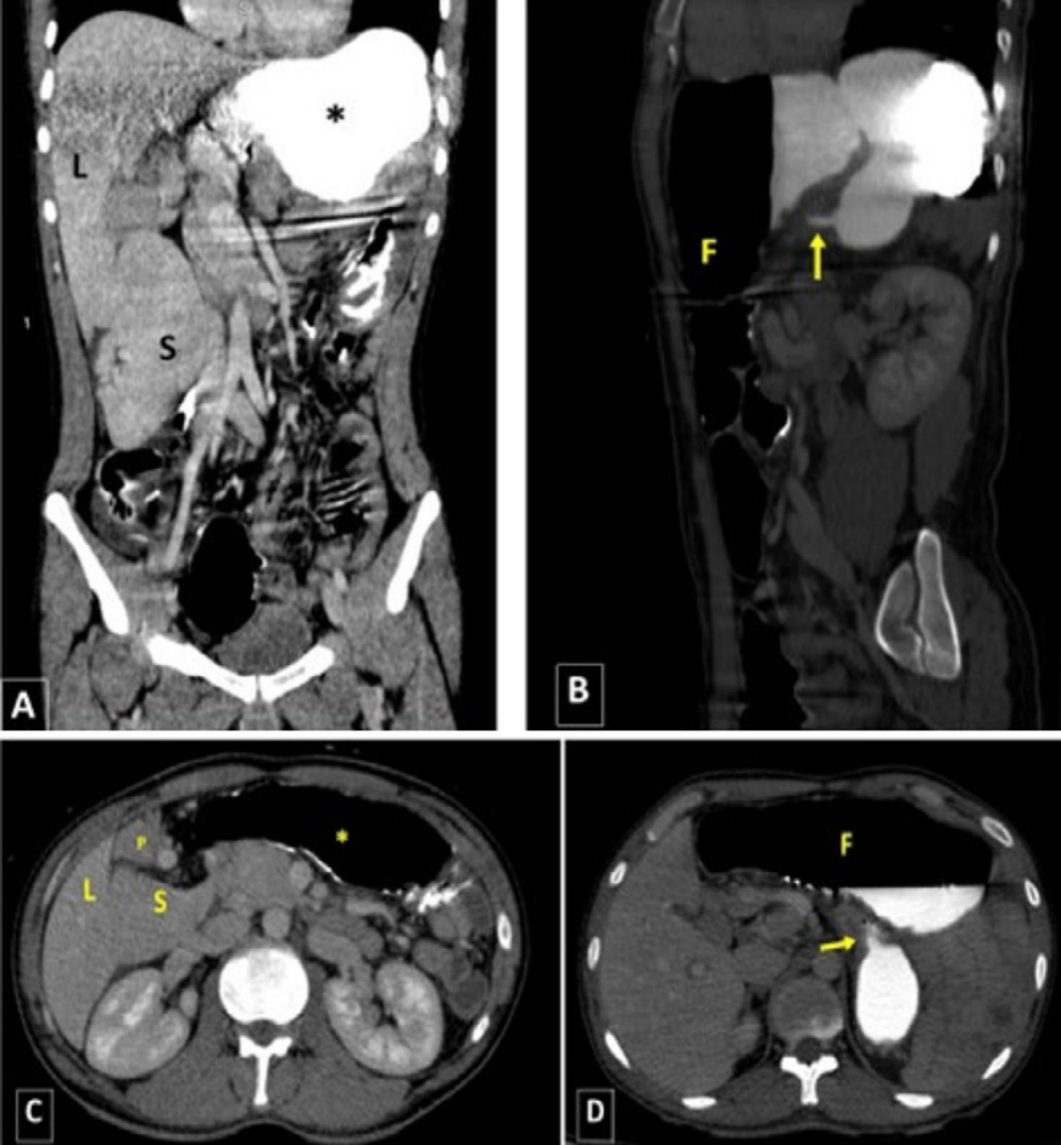
(A) Coronal and (C) axial planes of CECT demonstrating displacement of the spleen (S) and pancreas (P) to the right side of the abdomen. (B) Sagittal and (D) axial planes show malposition of the fundus (F), located anterior and inferior relative to the pylorus sphincter (arrow). Also, the antrum and pylorus were located on the left side. (L: Liver), (S: Spleen), (*: Stomach), (F: Fundus).

Accordingly, upright and supine abdominal X‐rays were requested (Figure 4), demonstrating evidence of gastric dilation. Due to the patient's frequent vomiting and food intolerance, an NG tube was inserted. For further evaluation, an abdominal radiograph with oral contrast was repeated, revealing abnormal stomach positioning with the fundus displaced to the anterior‐inferior region relative to its axis. Delayed imaging showed no passage of contrast medium from the stomach to the intestinal loops, raising strong suspicion for mesenteroaxial volvulus (Figure 5). Subsequently, the patient underwent a CT scan with contrast injection. In the CECT images, a severe gastric dilation with an abnormal axis was observed. Furthermore, gastric cardia was seen in the midline, whereas the antrum and pylorus were on the left side, posterior to the stomach and inferior to the gastroesophageal junction (GEJ). The distal loops of the duodenum and jejunum were also collapsed. Additionally, some abdominal organs were displaced, with the spleen located on the right side, near the lower and inner portion of the liver. The tail and body of the pancreas were also observed along the splenic vein, extending towards the splenic hilum in the subhepatic region. Moreover, evidence of total colectomy and mild free fluid in the abdominopelvic cavity was observed. These findings were consistent with a mesenteroaxial gastric volvulus alongside a wandering spleen and moderate pancreatic volvulus (Figure 6). The laboratory test results were as follows: WBC: 7600/μL, neutrophils: 62%, Hb: 13.5 g/dL, platelets: 398,000/μL, amylase: 73 U/L, lipase: 89 U/L, ALT = 25 U/L, AST = 31 U/L, ALP = 102 U/L, Na = 143 mEq/L, K = 4.6 mEq/L.

### 3.3 Conclusions and Results (Outcome and Follow‐Up)

Based on the overall findings, the patient underwent IV fluid resuscitation, IV antibiotic injection, Foley catheter fixation, and I/O charting in the emergency room before being transferred to the operating room for surgical intervention. There, a vertical midline laparotomy was performed, during which the surgeon repositioned the spleen and pancreas to the right side and rotated the stomach 180 degrees around the splenic mesentery. No signs of ischemia were observed. Following successful detorsion of the volvulus, both the stomach and spleen were fixed (gastropexy and splenopexy, respectively), and a gastrostomy was inserted. The postoperative course was uneventful, and the patient was discharged in good general condition after 13 days.

## 4 Discussion

Gastric volvulus is a rare yet serious gastrointestinal (GI) tract disorder commonly leading to obstructive conditions. Given its severity and diagnostic challenges, a comprehensive understanding of its clinical features, diagnostic methods, and management strategies is crucial for timely and effective intervention.

Gastric volvulus could be classified etiologically as primary or secondary, based on the axis of rotation as organoaxial or mesenteroaxial, partial or complete based on the severity of rotation, or complicated or uncomplicated according to the related consequences [2, 5].

As mentioned, gastric volvulus can be categorized according to the malrotation axis. The organoaxial (OA) volvulus involves the stomach rotating along its longitudinal axis, extending from the gastric inlet to the outlet, and is often likened to the motion of “wringing out a wet rag” [11]. In contrast, mesenteroaxial (MA) volvulus rotates around the transverse axis, which runs from the midpoint of the lesser curvature to the midpoint of the greater curvature [1]. MA volvulus is less common, tends to occur in children, and is often linked to congenital diaphragmatic hernias or other anatomical abnormalities [12]. OA occurs roughly twice as frequently as MA volvulus, while a mixed form is identified in approximately 10% of cases [1].

Moreover, a rotation exceeding 180° is classified as severe or complete and increases the risk of gastric outlet obstruction (GOO) and strangulation [13, 14]. Complete volvulus is more frequently associated with OA rotation and typically presents as a sudden narrowing, often described as having a bird‐beak appearance. In partial rotation, where the twist is less than 180°, a distinct transition point may be absent, allowing oral contrast to pass beyond the site of the volvulus. Partial volvulus can occur in both OA and MA types, though MA volvulus is more commonly partial [5].

The clinical presentation of gastric volvulus differs within a vast spectrum; however, the classic Borchardt's triad doesn't always exist. Other clinical presentations include intermittent dysphagia, abdominal distension, acid regurgitation, abdominal discomfort, early satiety, weight loss, and anemia [2, 5]. Given the diversity of the clinical presentation, the suspicion of gastric volvulus should always be confirmed using imaging modalities. The plain radiographs, chest or abdominal CT scans, and the upper GI series have the most expansive use among the various modalities. In contrast, endoscopy has limited reliability in diagnosing gastric volvulus, with a failure rate of around 68%. However, it remains helpful in distinguishing other potential conditions and for gastric decompression [15].

On plain radiographs, findings may include two distinct air‐fluid levels in the antrum and fundus, a solitary air bubble without additional luminal gas when the patient is supine, or a “beak” appearance near the cardio‐esophageal junction [8]. The upper GI series is typically not performed as the initial imaging method for patients suspected of gastric volvulus, as symptoms are often nonspecific, and the condition's rarity leads to low clinical suspicion [11, 16]. Instead, many patients, especially those presenting with acute abdominal pain, are more likely to undergo CT scans, where signs of gastric volvulus may be detected. However, gastric volvulus poses diagnostic challenges when using CT imaging due to its uncommon occurrence and similar appearance to large hiatal hernias [6]. Accordingly, Mazhaheri et al.'s investigation on CT imaging revealed that the most frequent and sensitive CT findings related to gastric volvulus were stenosis at the hernia neck (average sensitivity and positive likelihood ratio 79% and 19.7, respectively) and transition point at the pylorus (average sensitivity and positive likelihood ratio 75% and 16, respectively).

Blood tests could also raise suspicion of the related complications, as a raised WBC count and neutrophil ratio can indicate intestinal necrosis, while electrolyte imbalance may reflect the obstructive nature of gastric volvulus [17]. Additionally, peri‐gastric fluid accumulation or pleural effusion in the imaging could indicate tissue ischemia or necrosis [6].

The presenting paper described two cases of an extremely rare combination of gastric volvulus, wandering spleen, and pancreatic volvulus. In the first patient, the pancreatic volvulus even led to degrees of pancreatitis, as elevated amylase and lipase and the radiologic characteristics around the pancreas suggested. A wandering spleen happens when the spleen's suspensory ligaments are either loose or absent, causing the organ, typically fixed in the left upper quadrant, to move freely within the abdominal cavity [18]. Gastric and pancreatic volvuli and wandering spleen are all rare pathologies, and their coexistence is extremely rare, with limited evidence existing in the literature [19, 20]. In 2014, Gorsi et al. first described this triad as a rare cause of acute abdominal pain, requiring emergent surgical intervention [21]. From then on, there have only been a few reports of this scarce triad in the literature, sharing the same presentation of acute abdominal pain worsening over a short period of time. Nausea and bilious emesis, fever and chills, history of recurrent hospital admissions due to abdominal pain and pancreatic volvulus, severe epigastric or generalized tenderness on abdominal examination, leukocytosis, cholestasis and hyperbilirubinemia, and elevated liver or pancreatic enzymes were among the other findings in the literature. Considering the nonspecific clinical presentation and the rarity of this pathology, diagnosis can only be confirmed through radiologic examination, underscoring the importance of radiologists' awareness of this condition [22, 23, 24, 25]. In these cases, the wandering spleen can be treated with either splenopexy or splenectomy. If splenic ischemia occurs, splenectomy is the preferred option [26]. The displacement of organs is reported to be a rare but extremely dangerous complication of gastric volvulus. Besides increasing the risk of localized damage or dysfunction or the risk of strangulation, this could potentially result in a decreased cardiac output, which in turn could fatally compromise the cardiorespiratory function [1].

Initial management of gastric volvulus typically involves nasogastric decompression to relieve gastric distension and stabilize the patient's hemodynamic status [8]. Definitive treatment often requires surgical intervention to correct the volvulus and address any underlying anatomical defects. Surgical options include laparoscopic or open reduction of the volvulus, gastropexy to prevent a recurrence, and repair of associated hernias or diaphragmatic defects. In cases where the stomach is necrotic, partial or total gastrectomy may be necessary [2, 5, 8]. In summary, the most frequently performed procedure is the surgical reduction of the volvulus through laparotomy, either with or without gastropexy, intended for preventive purposes [8]. The prognosis of gastric volvulus largely depends on the timeliness of diagnosis and intervention. Delayed treatment can lead to severe complications and increased mortality rates, whereas early recognition and surgical correction increase the chance of favorable outcomes [2].

## 5 Conclusion

In conclusion, the triad of gastric volvulus, pancreatic volvulus, and wandering spleen represents an exceptionally rare but critical clinical entity requiring prompt recognition and intervention. Each component of this triad poses unique diagnostic and therapeutic challenges, and their coexistence further complicates clinical management. Early detection through imaging is crucial, as delays in diagnosis can lead to life‐threatening complications, including ischemia, obstruction, and organ necrosis. Surgical intervention remains the mainstay of treatment, tailored to the underlying anatomical abnormalities. A comprehensive understanding of this rare combination is essential for improving patient outcomes and guiding appropriate management strategies.

## Author Contributions

**Parvaneh Layegh:** resources, supervision, writing – review and editing. **Atiyeh Mahdavi Rafie:** project administration, writing – original draft, writing – review and editing. **Armin Doostparast:** writing – original draft, writing – review and editing.

## Consent

Written informed consent was obtained from the patient for publication of this report in compliance with the journal's patient consent policy.

## Conflicts of Interest

The authors declare no conflicts of interest.

## Data Availability

All data generated or analyzed in this study is presented within the manuscript.
